# Characterization of an Alkaline Alginate Lyase with pH-Stable and Thermo-Tolerance Property

**DOI:** 10.3390/md17050308

**Published:** 2019-05-24

**Authors:** Yanan Wang, Xuehong Chen, Xiaolin Bi, Yining Ren, Qi Han, Yu Zhou, Yantao Han, Ruyong Yao, Shangyong Li

**Affiliations:** 1 Department of Pharmacology, College of Basic Medicine, Qingdao University, Qingdao 266071, China; sunshine4581@163.com (Y.W.); chen-xuehong@163.com (X.C.); Renyn796@163.com (Y.R.); xiaoyu19990727@163.com (Q.H.); zy18339956716@163.com (Y.Z.); 2 Department of Rehabilitation Medicine, Qingdao University, Qingdao 266071, China; 18661809159@163.com; 3 Central Laboratory of Medicine, Qingdao University, Qingdao 266071, China; yry0303@163.com

**Keywords:** Alginate lyase, Thermo-tolerant, pH-stability, Endo-manner, *Vibrio* sp. SY01

## Abstract

Alginate oligosaccharides (AOS) show versatile bioactivities. Although various alginate lyases have been characterized, enzymes with special characteristics are still rare. In this study, a polysaccharide lyase family 7 (PL7) alginate lyase-encoding gene, *aly08*, was cloned from the marine bacterium *Vibrio* sp. SY01 and expressed in *Escherichia coli.* The purified alginate lyase Aly08, with a molecular weight of 35 kDa, showed a specific activity of 841 U/mg at its optimal pH (pH 8.35) and temperature (45 °C). Aly08 showed good pH-stability, as it remained more than 80% of its initial activity in a wide pH range (4.0–10.0). Aly08 was also a thermo-tolerant enzyme that recovered 70.8% of its initial activity following heat shock treatment for 5 min. This study also demonstrated that Aly08 is a polyG-preferred enzyme. Furthermore, Aly08 degraded alginates into disaccharides and trisaccharides in an endo-manner. Its thermo-tolerance and pH-stable properties make Aly08 a good candidate for further applications.

## 1. Introduction

Alginate is an acidic hetero-polysaccharide extracted from brown algae, which accounting for 22–44% of its dry weight [1,2,3]. Alginate mainly contains two different uronic acids, including α-l-guluronic acid (G) and β-d-mannuronic acid (M). They are arranged into three different kinds of blocks by (1→4)-linked monosaccharides: homopolymeric G blocks, polyguluronate (PolyG); homopolymeric M blocks, polymannuronate (PolyM); and random or heteropolymeric blocks of alternating M and G units (PolyMG) [4,5]. 

Alginate lyase (E.C. 4.2.2.3 and E.C. 4.2.2.11) is a kind of polysaccharide lyase that degrades alginate by β-eliminating the glycoside 1-4 O-bonds between C4 and C5 at the non-reducing end, thus producing unsaturated alginate oligosaccharides (UAOS) as main products [6,7]. Due to its high efficiency, specificity and mild degradation function, alginate lyases have attracted widespread attention in industrial applications, especially in the preparation of alginate oligosaccharides [8,9]. 

According to the Carbohydrate-Active enZYmes (CAZy) databases, alginate lyases belong to PL families 5, 6, 7, 14, 15, 17, and 18 based on the analysis of their amino acid sequences [10,11,12]. Based on the substrate specificity, alginate lyases can be further classified into two types, one type is the G block-specific lyases (polyG lyases, EC 4.2.2.11), and the other type is the M block-specific lyases (polyM lyases, EC 4.2.2.3) [13,14]. In PL families 5, 7, 14, 17, and 18, most of the reported alginate lyases are polyM lyases. Only the alginate lyase reported in PL family 6 is mainly comprised of polyG lyases. Thus far, hundreds of alginate lyases have been purified, cloned, and characterized from marine microorganisms, brown seaweeds, and mollusks [15,16,17,18]. However, these reported enzymes with characteristics specific for commercial use are rare. Cold-adapted alginate lyases can run biocatalytic processes at low temperature and reduce the danger of contamination. Thermo-tolerant enzymes persist at high temperatures, thereby not only improving degradation efficiency but also reducing production costs. Meanwhile, high proportion product in a mixture of products will be propitious to the purification of oligosaccharide. Therefore, there is an urgency to obtain an alginate lyase with the optimal characteristics (e.g., pH-stability, thermo-tolerance, and single product distribution) needed for industrial applications.

In this study, a new alginate lyase-encoding gene, *aly08*, was cloned from *Vibrio* sp. SY01, and expressed in *Escherichia coli* BL21 (DE3). The recombinant enzyme Aly08 degraded alginate, yielding alginate disaccharides and trisaccharides as main products. This study also revealed that Aly08 was a polyG-preferred enzyme with special characteristics, such as wide pH-stability, thermo-tolerance, and single product distribution. These special features suggest that Aly08 may play essential roles in saccharification processes of alginate and carbon cycling.

## 2. Results and Discussion

### 2.1. Sequence Analysis of Aly08

The marine bacterium *Vibrio* sp. SY01 was isolated from Yellow sea sediment, China. It grew rapidly in the alginate sole-carbon medium and efficiently degraded brown seaweed with a high alginate lyase activity (more than 50 U/mL). The genome sequence analysis of *Vibrio* sp. SY01 showed that it contained the putative alginate lyase-encoding gene, *aly08*, consisting of 897 bp of an open reading frame (ORF). The identified alginate lyase, Aly08, contained 299 amino acid residues. Signal peptide analysis showed that Aly08 predicted a putative signal peptide (Met^1^ to Phe^22^) in its N-terminal. Furthermore, the theoretically isoelectric point (pI) and theoretical molecular weight (Mw) of mature Aly08 were 4.57 and 32.89 kDa, respectively. According to a search of Conserved Domain Database (CDD) of NCBI, Aly08 is a new alginate lyase with a single-domain belonging to the alginate lyase superfamily 2.

**Figure 1:**
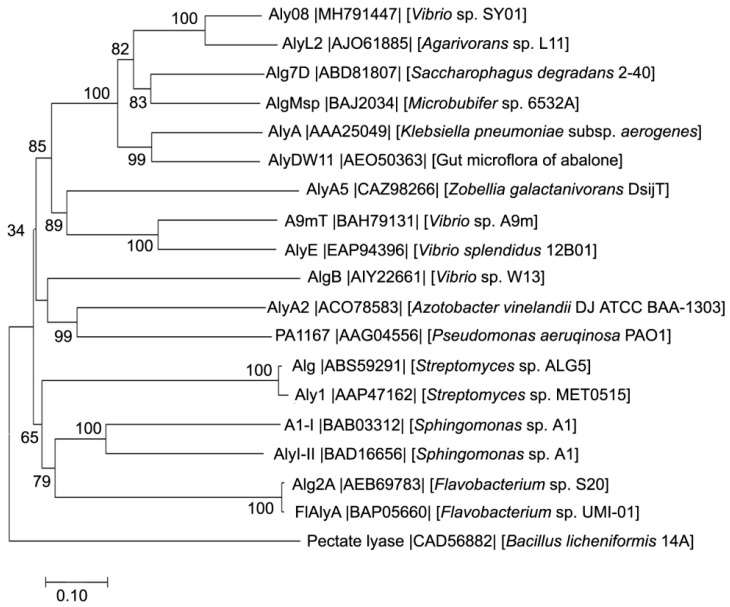
Phylogenetic analysis of Aly08 with other reported alginate lyases. The reliability of the phylogenetic reconstructions was determained by boot-strapping values (1000 replicates). Branch-related numbers are bootstrap values (confidence limits) representing the substitution frequency of each amino acid residue. A pectate lyase (CAD56882) from *Bacillus licheniformis* 14A was taken as control.

Based on the sequences of Aly08 and other reported PL family 7 alginate lyases, phylogenetic trees were created. Pectate lyase (Genbank number CAD56882) from *Bacillus licheniformis* 14A was included as a control (Figure 1). Among all of the reported alginate lyase, Aly08 had the highest identity sequence of amino acids (78%) with a PL family 7 alginate lyase, AlyL2 (Genbank number MH791447), from *Agarivorans* sp. L11 [19].

**Figure 2:**
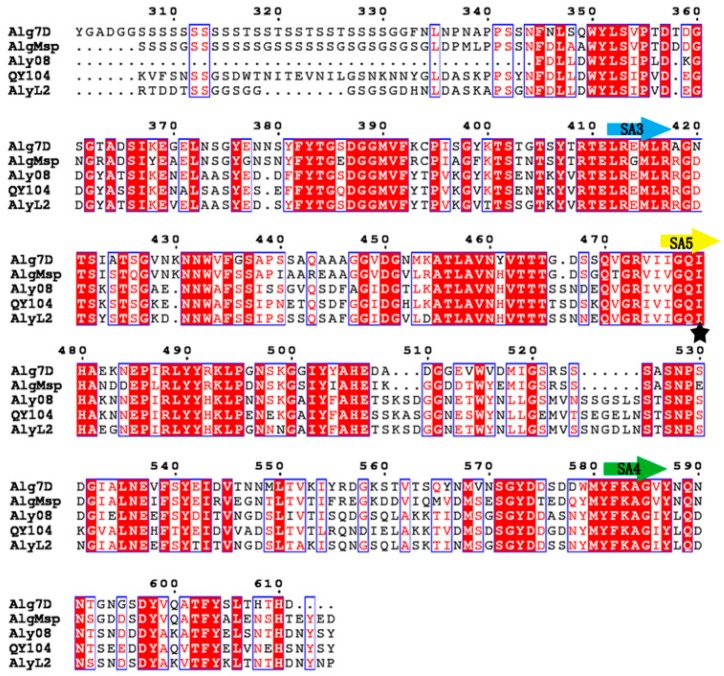
Sequence comparison of Aly08 with related alginate lyases from PL family 7: Alg7D (ABD81807) from *Saccharophagus degradans* 2–40, AlgMsp (BAJ62034) from *Microbulbifer* sp. 6532A, AlyV4 (AGL7859) from *Vibrio* sp. QY104, and AlyL2 (AJO61885) from *Agarivorans* sp. L11. The conserved regions and identical residues are marked with bands and black star, respectively.

A deeply branched cluster was formed in the phylogenetic tree among the enzymes Aly08 (Genbank number MH791447), AlyL2 (Genbank number AJO61885), AlgMsp (Genbank number BAJ62034), and Alg7D (Genbank number ABD81807). According to the multiple sequence alignment (Figure 2), the enzyme contains three conserved regions “RTELREMLR”, “QIH”, and “MYFKAG” which are related to substrate binding and catalytic activity in PL family 7 [20]. These results identified Aly08 as a new member of the PL family 7. Thus far, several alginate lyases have been identified from various bacteria, such as *Pseudoalteromona, Flavobacterium*, *Nitratiruptor*, *Agarivorans*, and *Vibrio* [7,17,21,22,23]. After determining their various properties, most of the alginate lyases showed a preference towards polyM blocks. In this study, the purified alginate lyase Aly08 is a polyG-preferred alginate lyase containing the “QIH” conserved region, according to their sequence analysis (Figure 2). The conserved region, “QIH” or “QVH”, plays a key role in the substrate preferences of alginate lyases. Aly08, along with AlgMsp, A1-II and Alg2A containing the “QIH” conserved region showed preferences for polyG [24,25,26]. In addition, other alginate lyases derived from PL family 7, such as AlxM from *Photobacterium* sp. ATCC 43367, and A9mT from *Vibrio* sp. A9m, possess “QVH” regions show preference for degrading polyM blocks (Table 1) [27,28]. Through further sequence screening, it was found that “QIH” or “QVH” may be an indicator for substrate-preferred analysis. Furthermore, Aly08 can be used for the next part of combining a polyM-preferred alginate lyase for synergetic degradation alginate or brown seaweeds.

### 2.2. Expression, Purification, and Characterization of Aly08

**Figure 3:**
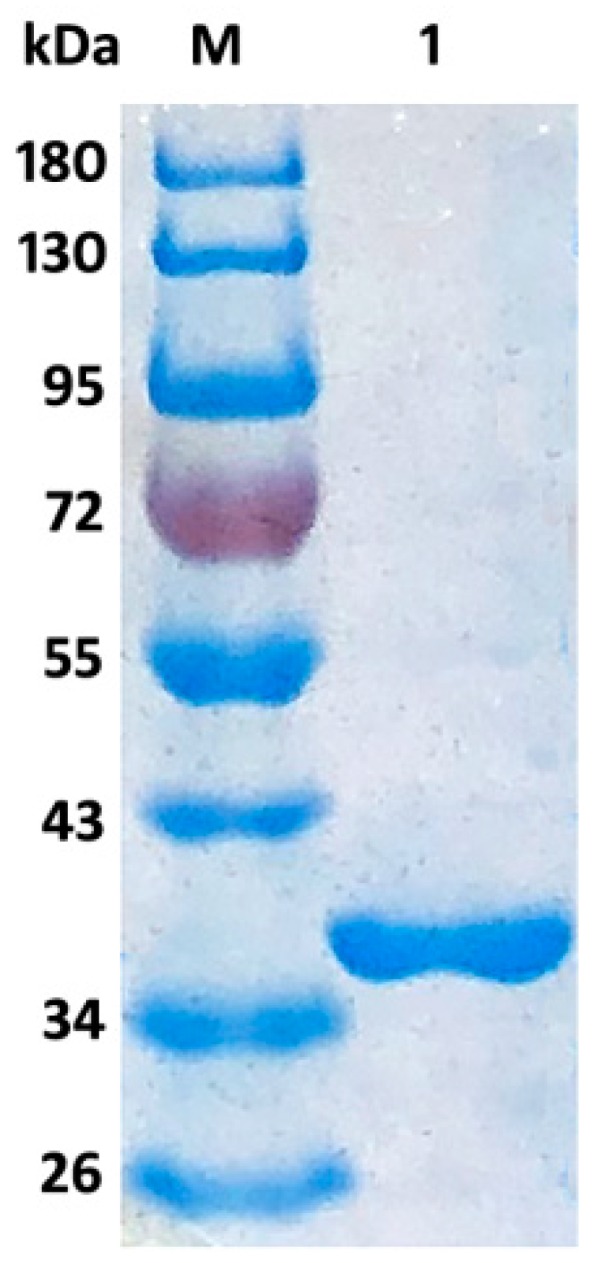
SDS-PAGE analysis of the recombinant enzyme Aly08. Lane M, protein marker; Lane 1, the purified Aly08.

The expression strain *E. coli* BL21-pET22b-Aly08 was grown in LB broth and Aly08 was purified by a Ni-NTA affinity column. The specific activity of purified Aly08 was 841.1 U/mg with high viscosity sodium alginate as substrate. Moreover, the purified enzyme Aly08 was analyzed by sodium dodecyl sulfate polyacrylamide gel electrophoresis (SDS-PAGE) and observed as a single band on the gel with an approximate Mw of 35 kDa (Figure 3), which was corresponding to the theoretical molecular mass of 32.89 kDa.

**Figure 4:**
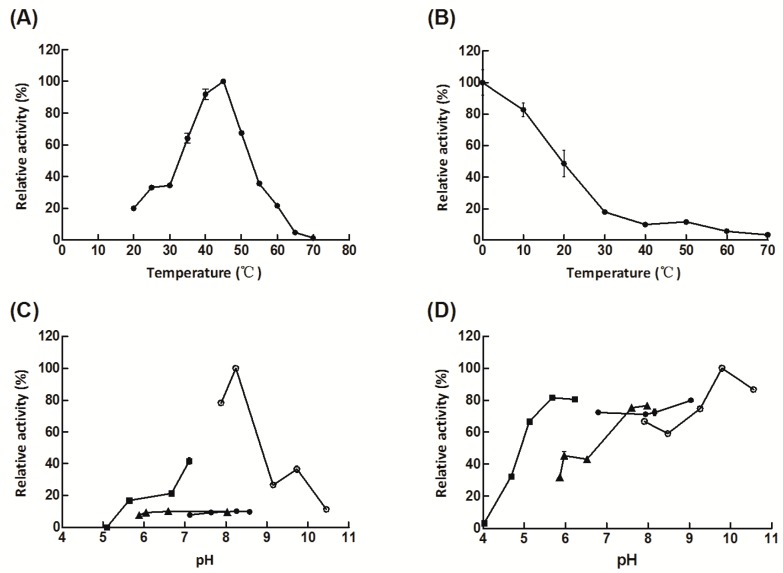
The biochemical characteristics of Aly08. (**A**) The optimal temperature of Aly08. (**B**) The thermal-stability of Aly08. (**C**) Optimal pH for the relative activity of Aly08 was determined in 20 mM Tris-HCl buffer (solid circle), 20 mM phosphate buffer (solid triangle), 20 mM citic-Na_2_HPO_4_ (solid square), or 20 mM glycine-NaOH buffer (hollow circle). (**D**) pH stability of Aly08 in 20 mM Tris-HCl buffer (solid circle), 20 mM phosphate buffer (solid triangle), 20 mM citic-Na_2_HPO_4_ (solid square), or 20 mM glycine-NaOH buffer (hollow circle).

Then, the characterization of purified Aly08 was analyzed as follows. The enzyme Aly08 showed maximum activity at 45 °C, and maintained activities of 82.8% and 48.7% when the enzyme was incubated at a low temperature for 1 h, 10 °C and 20 °C, respectively (Figure 4A,B). The optimal pH for Aly08 was found to be 8.35 (Figure 4C). In addition, Aly08 holds more than 60% of activity in a wide pH range from 7.0–11.0 after incubation in different buffers at 4 °C for 12 h, and was particularly stable under alkaline conditions. (Figure 4D). As previous study, most of the alginate lyases showed optimal pH and stability close to a neutral environment, such as AlyH1 from *Vibrio furnissii* H1 shows high activity at the optimal pH 7.5 and it was stable at pH 6.5–8.5. In addition, AlyH1 only retains about 20% of residual activity when incubated at pH 9.5 for 12 h [35]. AlgNJU-03 from *Vibrio* sp. NJU-03 possessed a neutral optimal pH at 7.0 and its pH-stability range from 6.0 to 8.0 [20]. Those alginate lyases prefer neutral pH and they only show high activities within a narrow pH range after incubating for several hours and always exhibit instability under alkaline conditions. Another reported high-alkaline alginate lyase A1m from marine bacteria *Agarivorans* sp. JAM-A1m, exhibited high activity at pH 9.0 under glycine-NaOH buffer with 0.2 M NaCl added to the reaction mixture. However, A1m was only stable and maintain more than 60% of residual activity in a short period of 1 h over a narrow pH range of 7.0–9.0 [33]. Comparing with A1m, Aly08 was stable over 12 h with its 80% residual activity even at pH 9.0–11.0, while another enzyme derived from *Vibrio* sp. NJ-04 maintains good stability at pH 4.0–10.0, but the enzyme only maintains its maximum activity under neutral conditions (pH 7.0) [36]. Thus, Aly08 is an alkaline-stable lyase with industrial application potential as it has been proven to conduct catalysis reactions and maintain activity in a broader pH range.

In particular, the substrate preferred by Aly08 was determined by experimenting with three polymeric substrates (sodium alginate, polyM block and polyG block). Aly08 was found to prefer polyG blocks (1078.2 U/mg) rather than polyM blocks (297.1 U/mg) and native alginate (841.1 U/mg).

Moreover, activities of Aly08 were enhanced by NaCl (different concentrations from 10 mM to 3 M), and the activity reached its maximum at 300 mM NaCl, at which point the activity was about eight times higher than the activity in the absence of NaCl (Table 2). Similarly, the activity of AlgM4 from *V. weizhoudaoensis* M0101 was increased about seven times at 1 M NaCl and activated by a concentration range of NaCl at 0–1 M [37]. For these alginates from marine bacteria, a certain level of NaCl concentration is essential for strain survival and enzyme activation. The effects of other metal ions on the activity of Aly08 were also shown in Table 2. Aly08 showed no obvious activated effect in the presence of NH_4_^+^, Li^+^, Zn^2+^, Ba^2+^, and Co^2+^, while Al^3+^ and Ni^2+^ showed no obvious inhibiting effects on relative enzymatic activity. Enzyme activity was activated by divalent ions, such as Ca^2+^ and Mn^2+^. Different concentrations of KCl had little effect on the activities of Aly08 (Table S2). Interestingly, the enzyme activity of the reaction system containing divalent ions of Ca^2+^ was about twice as high as that of the reaction system without any ions. However, other reagents such as SDS and EDTA showed significant inactivation effects wherein relative activity was reduced to 50% and 55.9%, respectively.

### 2.3. Thermo-Tolerance and Heat Recovery of Aly08

**Figure 5:**
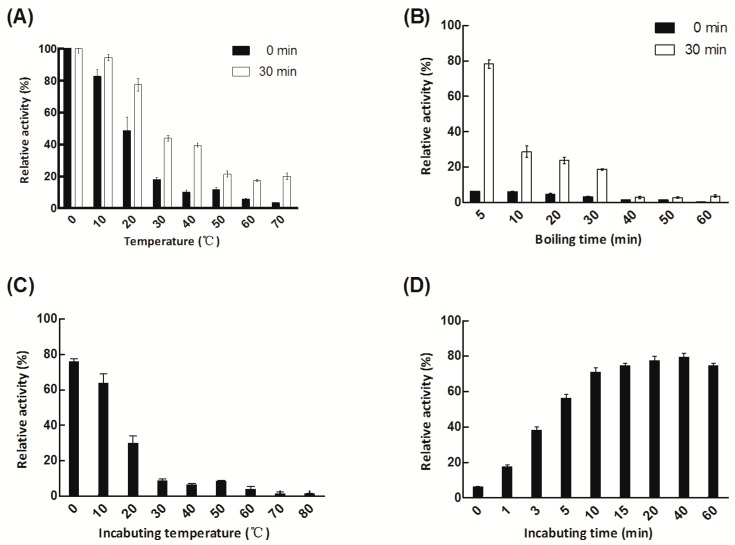
Thermo-tolerance and heat recovery of Aly08. (**A**) The difference of thermostability of enzymes incubation at ice-bath for 0 min (black columnar) and 30 min (white columnar). (**B**) Effects of boiling times on enzyme Aly08. Black and white columns indicate the activity of the heat-inactivated enzyme following ice-bath for 0 min and 30 min, respectively. (**C**) Effects of different incubation temperatures on the activity recovery of Aly08 under 5 min heat-inactivated conditions. (**D**) Effects of incubation time at 0 °C on the activity recovery of heat-inactivated Aly08. The enzyme activity without any treatment was 100%.

When we sought to determine the thermostability of Aly08, we found an interesting phenomenon. After ice-bath, the residual activity of heat-treatment enzymes was always higher than that of enzymes without an ice-bath (Figure 5A,B). After incubation at 30 °C and 40 °C for 1 h, Aly08 retained only 17.9% and 9.9% of its initial activity when directly assayed its activities. However, when the enzyme incubation at 0 °C for 30 min, the residual activity could recover to 43.8% and 39.4% in the same heat treatment condition (Figure 5A). Moreover, even the enzyme was boiled for 5 min, Aly08 was able to recover 78.3% of its initial activity after 30 min incubating in the ice-bath (Figure 5B).

To determine the optimal incubation temperature that contributed to the recovery of activity after boiling for 5 min, the enzyme was incubated for 30 min at various temperatures (0–80 °C). The recovered activity of the enzyme reached levels of 76.3%, 63.9%, and 30.1% after incubation at 0 °C, 10 °C, and 20 °C for 30 min, respectively. When the incubation conditions were above 30 °C, the recovery of activity was measured at less than 10% (Figure 5C). 

To further determine the optimal incubation time that contributed to the recovery of activity, the enzyme was incubated at 0 °C for different times. The activity of Aly08 gradually increased with prolonged culture time at 0 °C. Aly08 was rapidly re-activated approximately 56.7% and 71.3% of its activities after incubation at 0 °C for 5 min and 10 min, respectively. After incubation for 20 min, the activity was restored to 77.9%, after which the activity recovery rate began to decrease (Figure 5D).

The thermo-stability experiment indicated that low temperature may contribute to the recovery of Aly08. The thermo-tolerance of Aly08 could promote effective storage and transportation as the inactivated enzyme with heat treatment is able to successfully restore most of its activity after incubation at 0 °C.

### 2.4. Action Pattern and Final Product Analysis

**Figure 6:**
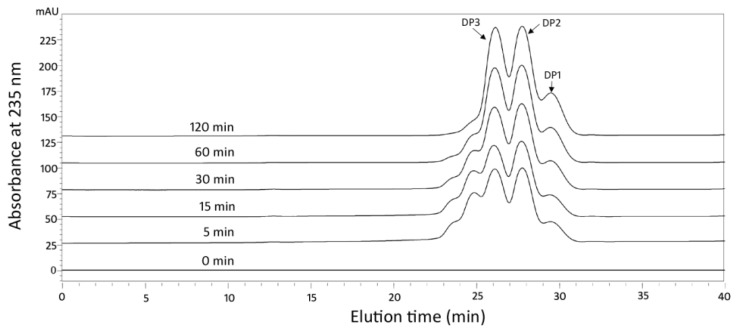
Degradation patterns of Aly08 toward sodium alginate. The elution positions of the unsaturated oligosaccharide product fractions with different degrees of polymerization are shown with arrows: DP1 represents unsaturated monosaccharide, DP2 represents unsaturated disaccharide, DP3 represents unsaturated trisaccharide.

The action mode of Aly08 was determined by size-exclusion chromatography with a Superdex^TM^ peptide 10/300 column (General Electric Company, Boston, MA, USA) using high-performance liquid chromatography (HPLC) platform (Figure 6). The hydrolysis pattern of Aly08 works as an endo-type because of the rapid depolymerization of substrates, the rise in polydispersity, and the production of intermediate oligosaccharides. Meanwhile, the action mode of Aly08 was further monitored by viscosity analysis (Figure S1). The viscosity of the alginate solution decreased rapidly during the first 5 min following the addition of Aly08 but changed little during subsequent time periods. During the whole observation period, the oligosaccharide content which was tested by A235 increased steadily. It can be further suggested that Aly08 is an endo-type enzyme in accordance with this finding.

**Figure 7:**
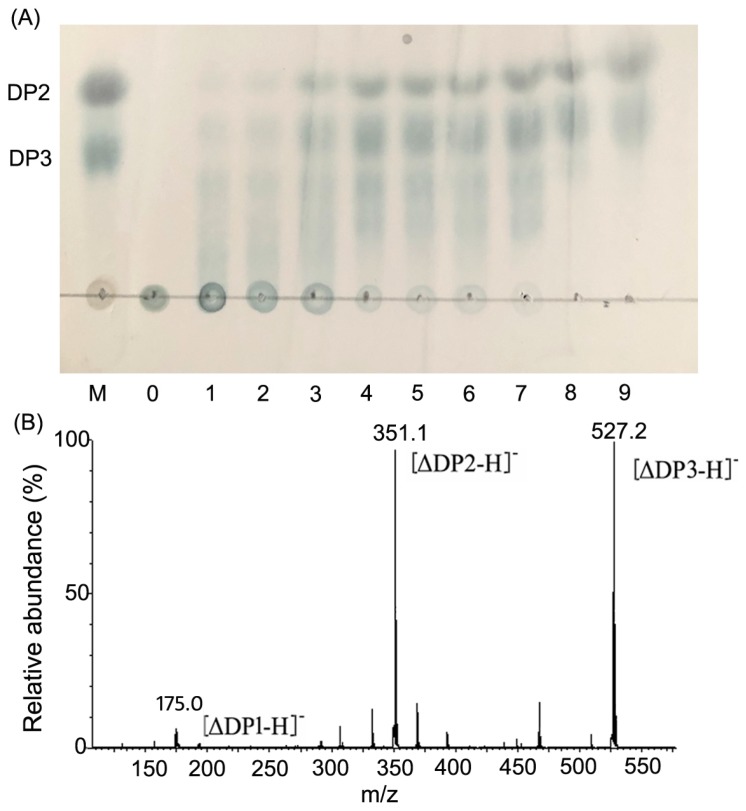
The hydrolytic products of Aly08. (**A**) TLC analysis of the hydrolytic products of Aly08. *Lane M*, standard alginate oligosaccharides (DP2-3); Line 0, alginate; Lane 1–9, hydrolytic products of Aly08 for different times (1, 2, 5, 10, 15, 30, 60, 90, and 120 min) toward 0.3% (*w*/*v*) high viscosity sodium alginate. DP2 and DP3 indicate alginate disaccharide and trisaccharide, respectively. (**B**) ESI-MS analysis of the end products of Aly08.

The hydrolytic degradations were analyzed by thin-layer chromatography (TLC) method after the alginate was completely degraded (Figure 7A). In the hydrolysis proceeds, there was a gradual decrease of alginate polysaccharide and an accumulation of oligosaccharides with various DPs. And two clear spots of end product (2 h) appeared on the TLC plate, indicating that the migration rate was in good agreement with the alginate disaccharide (DP2) and trisaccharide (DP3) marker. The final degradation product was also determined by negative-ion electrospray ionization mass spectrometry (ESI-MS) (Figure 7B). Two main spectra were 351.1 *m/z* [ΔDP2-H]^−^ and 527.2 *m/z* [ΔDP3-H]^−^, corresponding to the molecular mass of the unsaturated alginate disaccharides and trisaccharides, respectively [38].

Through HPLC, viscosity, TLC and ESI-MS analysis, Aly08 was shown to degrade alginate polymer as an endo-type manner, eventually degrading alginate into disaccharides and trisaccharides. Previous studies have reported that enzymatic oligosaccharide products have a variety of specific biological activities and possess broad potential application prospects in many fields, such as antioxidant activities, regulation of plant root growth, anti-inflammatory activities, and reguation of lipoprotein metabolism [39,40,41,42]. The single homogeneous products in the progress of enzymatic production of oligosaccharides is conducive to the oligosaccharide purification and application. Thus far, most of the reported products of alginate lyase are mixtures of DP2–DP5, such as AlyA-OU02 from *V. spiendidus* OU02, appear to take disaccharides, trisaccharides, and tetrasaccharides as the main hydrolytic products [28]. Additionally, the final degradation products of the alginate lyase FlAlyA from *Flavobacterium* sp. UMI-01 are DP2-DP5 [34]. Compared with those alginate lyase, the end products of Aly08 are a disaccharide and trisaccharide, which are advantageous for further separation and industrial high-efficiency production. Aly08 may have potential as a tool for the preparation of single homogeneous products of monosaccharides which have wide pharmaceutical applications.

## 3. Materials and Methods

### 3.1. Materials

High viscosity sodium alginate (20–50 kDa, 100–260 monosaccharide in polymer, M/G ratio: 1.66) and low viscosity sodium alginate (1–5 kDa, 5–26 monosaccharide in polymer the, M/G ratio: 1.66) was purchased from Bright Moon Seaweed Group (Qingdao, China), PolyM and PolyG blocks (purity: 95%) were purchased from Qingdao BZ Oligo Biotech Co., Ltd. (Qingdao, China). Standard alginate disaccharide and trisaccharide were also purchased from Qingdao BZ Oligo Biotech Co., Ltd. Standard monosaccharide (glucuronic acid) was purchased from Sigma. In addition, *E. coli* strains DH5α and BL21 (DE3) (Solarbio, Beijing, China) were grown in Luria–Bertani (LB) medium and used for plasmid construction and as a host for gene expression, respectively. LB broth supplemented with ampicillin (50 μg/mL) was used to grow both strains at 37 °C. The expression vectors used for gene cloning were pET-22b(+) (Novagen, Madison, WI, USA) plasmids. Oligonucleotides used for the cloning and expression of *aly08* were shown in Table S1.

### 3.2. Strains and Nucleotides

The sea mud samples were isolated from the sediment surface layer of Yellow Sea bottom (depth 36 m, E 120.13° N 35.76°, collected in May, 2017) and then immersed, diluted and spread on alginate sole-carbon selective medium plates [2 g (NH_4_)_2_SO_4_, 30 g NaCl, 0.1 g MgSO_4_·7H_2_O, 7 g K_2_HPO_4_, 3 g KH_2_PO_4_, 0.1 g FeSO_4_, 5 g sodium alginate, dissolved in 1 L distilled water with 10 g agar, pH 7.0)]. At least 100 strains were isolated from the detectable colonies after incubation at 25 °C for 4 days, and then the strains were inoculated into agar-free selective medium for the purpose of identifying the activities of alginate lyases. A higher activity strain was screened out, 27F and 1492R primers were used to amplify the 16S rDNA gene of this strain. The 16S rDNA gene sequence of this strain was blasted to obtain the closely related sequence using the BLASTn algorithm program, the sequence was aligned with its closely related sequences using MEGA 6.0. According to the sequence alignment, this strain was classified as *Vibrio* sp. SY01. This strain has been preserved at the China Center for Type Culture Collection (CCTCC) under no. M2018769. 

### 3.3. Sequence Analysis

In our previous study, genomic sequence analysis of *Vibrio*. sp SY01 showed a putative alginate lyase-encoding gene, *aly08*. The gene was cloned from the genome of strain SY01 and deposited in the Genbank database (accession number MH791447). The open reading frame (ORF) was identified with the program ORF finder (https://www.ncbi.nlm.nih.gov/orffinder/) and the signal peptide was analyzed using the SignalP 4.0 server (http://www.cbs.dtu.dk/services/SignalP/). The domain analysis of *aly08*, and its family analysis, is based on a comparison with the CDD (https://www.ncbi.nlm.nih.gov/cdd). The theoretically pI and Mw of *aly08* was determined by pI/Mw Tool (http://web.expasy.org/compute_pi/). Afterwards, the BLAST algorithm program on NCBI was used to search for similar sequences of *aly08*. Multiple sequence alignment was constructed using ClustalX 2.1 (National Center for Biotechnology Information, Bethesda, MD, USA), and the phylogenetic tree was created using the bootstrapping neighbor-joining method of MEGA 6.0 (Center for Evolutionary Medicine and Informatics, The Biodesign Institute, Tempe, AZ, USA).

### 3.4. Heterologous Expression and Purification of Recombinant Aly08

In order to express Aly08, the primers (PyAly08-F and PyAly08-R) were used to amplify the genomic DNA of *Vibrio* sp. SY01 without a signal sequence or stop codon. The *aly08* gene was then ligated into the expression vector pET-22b(+) with recognition sites *Nde* I and *Xho* I. In addition, the recombinant plasmid pET22b-Aly08 with a C-terminal 6 × His-tag was transformed into the *E. coli* BL21 (DE3) and grown on media (50 μg/mL ampicillin). Single colonies of *E. coli* BL21-pET22b-Aly08 were picked and cultured in LB medium (50 μg/mL ampicillin) at 37 °C with shaking at 200 rpm until OD_600_ reached 0.6–0.8. In order to induce the expression of protein, and the incubation was continued for 20 h at 20 °C (containing 0.1 mM isopropyl β-d-thiogalactoside (IPTG)). The cultured supernatant was harvested by high-speed refrigerated centrifuge (Hitachi, Tokyo, Japan) system (12,000 rpm, 5 min, 4 °C) and loaded onto a Ni-NTA sepharose column (GE Healthcare, Little Chalfont, Buckinghamshire, UK) using the AKTA150 automatic intelligent protein purification system (GE Healthcare, Little Chalfont, Buckinghamshire, UK) which had been equilibrated with wash buffer (20 mM phosphate buffer (pH 7.6), 500 mM NaCl). The Ni-NTA sepharose column was then eluted with a linear gradient of imidazole (25-500 mM imidazole, 20 mM phosphate buffer, 500 mM NaCl, pH7.6) in order to collect the active fractions. The active fraction was further analyzed by 12% SDS-PAGE, and the PageRuler Prest Protein Ladder (Thermo Scientific, USA) was used as a protein standard marker. Afterwards, the protein concentration of purified Aly08 was determined by a BCA protein assay kit (Beyotime Biotechnology, Shanghai China), bovine serum albumin (BSA) was used as a standard.

### 3.5. Alginate Lyase Activity Assay

Absorption at 235 nm (A235) was used to measure the activity of Aly08 as previously described [43,44,45]. The appropriately diluted enzyme (100 µL) was mixed with 900 µL of 0.3% (*w*/*v*) sodium alginate solution (10 mM glycine-NaOH buffer and 100 mM NaCl, pH 8.35). Then, the reaction system was incubated at 45 °C for 10 min and terminated by boiling for 10 min, and its absorbance was measured on a NanoPhotometer Pearl-360 spectrophotometer (IMPLEN, Munich, Germany). Alginate lyase activity was determined by increasing A235 as the production of unsaturated double bonds occurred as the alginate lyase cleaved glycosidic bonds at the non-reducing end of the polymer chain. One unit (U) of enzyme activity was defined as the amount of enzyme required to increase A235 by 0.1 per minute, under the above conditions.

### 3.6. Biochemical Characterization of the Recombinant Enzyme

The enzyme and substrate was incubated under 10 mM glycine-NaOH buffer (pH 8.35) at various temperatures (10–70 °C) to obtain the optimal temperature for Aly08. The thermal stability of Aly08 was then assayed by measuring its activity after pre-incubation at various temperatures (10–70 °C) for 1 h. The influence of pH values on Aly08 was calculated by measuring the residual activities in different buffers. The following buffers were used: 50 mM Na_2_HPO_4_-citric acid (pH 4.6–7.0), Tris-HCl (pH 7.6–8.6), glycine-NaOH (pH 8.6–10.6), and Na_2_HPO_4_-NaH_2_PO_4_ (pH 6.6–7.6). The highest activities represent 100% enzyme activity. The pH stability of Aly08 was determined by measuring the residual activity after incubating the purified enzyme with various pH buffers (pH 4.6–10.6) at 4 °C for 12 h. Substrate solution (10 mM glycine-NaOH buffer and 100 mM NaCl, pH 8.35) with three different substrates [0.3% (*w*/*v*) sodium alginate, polyM block and polyG block] were prepared and used for assaying the activities of the purified enzyme Aly08 in order to determine the preferred substrate of Aly08. Afterwards, based on the protein concentration measured by the BCA protein kit, the specific activity of Aly08 with the different substrates was calculated. To measure the effects of chemical compounds and metal ions on enzymatic activity, different metal ions and chemical compounds were added to the reaction system with a final concentration of 1 mM. A reaction mixture containing no metal ion or chemical compounds was used as a control. All reactions were performed in triplicate and the reaction parameters were expressed as the mean ± standard deviation.

### 3.7. Thermo-Tolerance Properties of Aly08 

Purified Aly08 was placed at different temperatures for 1 h and then divided into two parts. One part was directly measured for its activity, while the other part was measured for its activity following incubation in an ice-bath for 30 min. In order to further observe whether the difference in temperature stability is related to boiling time, different boiling times (5, 10, 20, 30, 40, 50, and 60 min) were selected to evaluate the residual activity of the purified enzyme. After that the heat-treatment group was divided into two parts, one part was directly measured for its activity, while the other part was measured for its activity following incubation in an ice-bath for 30 min. To determine the effects of different temperatures on the activity recovery of heat-inactivated Aly08, the enzyme was immediately incubated for 30 min between 0 °C and 80 °C after boiling 5 min. Moreover, the enzyme was boiled for 5 min and further incubated at 0 °C for different times (0–60 min) to evaluate the effect of the time of low temperature treatment on the recovery of enzyme activity after it was heat treated.

### 3.8. Analysis of Reaction Products and Hydrolytic Pattern

Low viscosity sodium alginate was used for analysis the reaction product of Aly08. In order to examine the hydrolysis pattern of Aly08, the products from different incubation times (0, 5, 15, 30, 60, and 120 min) were monitored at A235 using gel filtration chromatography with a Superdex^TM^ peptide 10/300 GL column with 0.2 M NH_4_HCO_3_ (flow rate: 0.6 mL/min) as an eluent on HPLC platform (LC-20A, Shimadzu, Japan) [46]. The mode of action was also analyzed using an Ostwald viscometer (No.1; Shibata Scientific Technology) with high viscosity sodium alginate as substrate. The equal component products (0.5 mL), which were degraded by Aly08 at 1, 5, 10, 15, and 30 min, were removed to characterize the viscosity and degradation products.

The hydrolytic degradation products were monitored using TLC method, wherein a reaction system containing 0.3% of high viscosity sodium alginate and Aly08 was constructed and the samples selected at different times (1, 2, 5, 10, 15, 30, 60, 90, and 120 min). The reaction products were analyzed using a TLC plate (TLC silica gel 60 F254, Merck KGaA, 64271 Darmstadt, Germany) with butanol/acetic-acid/water (2:1:1, by vol.) and color-developed with sulfuric acid/ethanol reagent (1:4, by vol.) after heating the TLC plate at 80 °C for 30 min. ESI-MS system (Thermo Fisher Scientific^TM^ Q Exactive^TM^ Hybrid Quadrupole-Orbitrap^TM^, Waltham, MA, USA) was employed to further investigate the composition and degree of polymerization (DP) of the end products.

## 4. Conclusions

In conclusion, we purified and characterized a new alginate lyase, Aly08, from marine bacterium *Vibrio* sp. SY01. Its special characteristics (such as: thermo-tolerance and pH stability) make Aly08 a superior candidate for industrial applications. Further analysis will focus on analyzing the three-dimensional structure of Aly08 and exploring its molecular mechanisms.

## Figures and Tables

**Table 1:** Comparison of the properties of Aly08 with other alginate lyases.

Protein Name	Optimal pH/Temperature (°C)	Conserved Region QIH/QVH	Substrate Specificity	Products (DP)	Source	References
Aly08	8.35/45	QIH	PolyG	2,3	*Vibrio* sp. SY01	This study
AlgNJU-03	7.0/30	QIH	PolyG, polyM,alginate	2,3,4	*Vibrio* sp. NJU-03	[20]
AlgMsp	8.0/40	QIH	PolyG	2–5	*Microbulbifer* sp. 6532A	[24]
A1-II’	7.5/40	QIH	polyG,polyM	3,4	*Sphingomonas* sp.A1	[29]
Aly2	6.0/40	QIH	polyG	2,3,4	*Flammeovirga* sp. strain MY04	[30]
AlyPI	7.0/40	QIH	polyG,polyM	-	*Pseudoalteromonas* sp. CY24.	[31]
Alg2A	8.3/40	QIH	polyG	5,6,7	*Flavobacterium* sp. S20	[26]
AlxM	-	QVH	polyM	-	*Photobacterium* sp. ATCC 43367	[32]
A1m	9.0/30	QIH	polyG	-	*Agarivorans* sp. JAM-A1m	[33]
A9mT	7.5/30	QVH	polyM	-	*Vibrio* sp. A9m	[27]
FlAlyA	7.7/55	QIH	polyM, polyG	2–5	*Flavobacterium* sp. strain UMI-01	[34]
AlyH1	7.5/40	QIH	polyG, alginate	2,3,4	*Vibrio furnissii* H1	[35]

**Table 2:** Effects of metal ions, EDTA and SDS on the activity of Aly08. Notes: Activity without addition of chemicals was defined as 100%. Data are shown as means ± SD (*n* = 3).

Reagent Added	Concentration (mM)	Relative Activity (%)
None	-	100.00 ± 0.24
	10	382.22 ± 2.64
NaCl	50	580.89 ± 4.36
	300	865.96 ± 26.46
	800	647.33 ± 11.25
	3000	361.97 ± 10.74
SDS	1	50.00 ± 3.32
EDTA	1	55.88 ± 8.60
Al_2_(SO_4_)_3_	1	85.33 ± 10.63
KCl	1	99.67 ± 0.86
KCl	100	103.54 ± 0.16
NiCl_2_	1	97.24 ± 2.20
(NH_4_)_2_SO_4_	1	105.56 ± 1.37
MnSO_4_	1	100.64 ± 1.78
Li_2_SO_4_	1	103.91 ± 0.93
ZnCl_2_	1	114.27 ± 2.48
BaCl_2_	1	120.74 ± 17.14
CoCl_2_	1	130.86 ± 4.03
MnCl_2_	1	166.39 ± 5.47
CaCl_2_	1	281.18 ± 29.18

## Supplementary Materials

The following are available online at https://www.mdpi.com/1660-3397/17/5/308/s1, **Figure S1.** Viscosity measurement of Aly08; **Table S1.** Primers used in this study.
