# Supplementary material for: Characterization of an Alkaline Alginate Lyase with pH-Stable and Thermo-Tolerance Property
Source: Mar Drugs. 2019 May 24;17(5):308. doi: 10.3390/md17050308 (PMC6562718; doi:10.3390/md17050308)
Supplement: Supplementary file 1 [file marinedrugs-17-00308-s001.pdf]

Supplementary Materials: Cloning and characterization of an alkaline alginate lyase with pH-stable and thermo-tolerance property

Yanan Wang, Xuehong Chen, Xiaolin Bi, Yining Ren, Qi Han, Yu Zhou, Yantao Han , Ruyong Yao and Shangyong Li

Table S1 Primers used in this study

Primers	sequences
PyAly08-F	5′- GGGAATTC CATATG ATGTTATTGAACAAAATTATT-3′
PyAly08-R	5′- CCG CTCGAG GTAAGAGTAGTTGTCGTGAGT-3′

Table S2 Effects of KCl of Aly08. Notes: Activity without addition of chemicals was defined as 100%. Data are shown as means ± SD (n = 3).

**Reagent added**	**Concentration (mM)**	**Relative activity(%)**
None	-	100.00±0.24
	1	99.67±0.86
KCl	10	100.32±1.38
	50	103.35±0.75
	100	103.54±0.16
	200	105.87±0.60

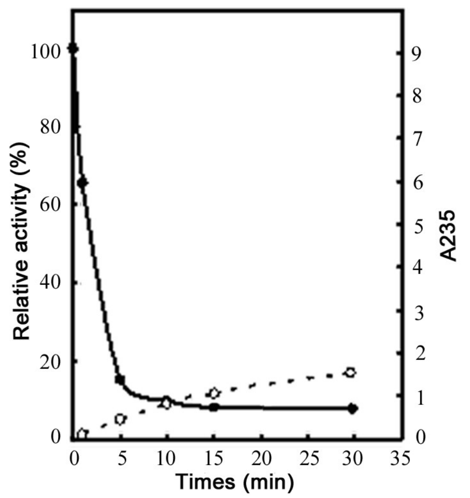

Fig S1 Viscosity measurement of Aly08. The initial viscosity of the reaction mixture without enzyme was taken as 100%. Rate of viscosity reduction is shown as open circles with solid line; the absorbance at 235 nm is shown as filled circles with dotted line
